# Genetic variation of male reproductive success in a laboratory population of *Anopheles gambiae*

**DOI:** 10.1186/1475-2875-6-99

**Published:** 2007-07-30

**Authors:** Maarten J Voordouw, Jacob C Koella

**Affiliations:** 1 Division of Biology, Imperial College of London, Silwood Park Campus, Buckhurst Road, Ascot, Berkshire, SL5 7PY, UK; 2 School of Life Sciences, Huxley Building, Keele University, Staffordshire, UK

## Abstract

**Background:**

For Anopheline mosquitoes, the vectors of human malaria, genetic variation in male reproductive success can have important consequences for any control strategy based on the release of transgenic or sterile males.

**Methods:**

A quantitative genetics approach was used to test whether there was a genetic component to variation in male reproductive success in a laboratory population of *Anopheles gambiae*. Swarms of full sibling brothers were mated with a fixed number of females and their reproductive success was measured as (1) proportion of ovipositing females, (2) proportion of ovipositing females that produced larvae, (3) proportion of females that produced larvae, (4) number of eggs laid per female, (5) number of larvae per ovipositing female and (6) number of larvae per female.

**Results:**

The proportion of ovipositing females (trait 1) and the proportion of ovipositing females that produced larvae (trait 2) differed among full sib families, suggesting a genetic basis of mating success. In contrast, the other measures of male reproductive success showed little variation due to the full sib families, as their variation are probably mostly due to differences among females. While age at emergence and wing length of the males were also heritable, they were not associated with reproductive success. Larger females produced more eggs, but males did not prefer such partners.

**Conclusion:**

The first study to quantify genetic variation for male reproductive success in *A. gambiae *found that while the initial stages of male reproduction (i.e. the proportion of ovipositing females and the proportion of ovipositing females that produced larvae) had a genetic basis, the overall reproductive success (i.e. the mean number of larvae per female) did not.

## Background

In the struggle against malaria, releasing genetically resistant mosquitoes is being given considerable attention as a potential means for future control [1,2]. Given that any control programme will most likely release transgenic males, the success of such a strategy depends on the reproductive fitness of these individuals. The past failures of releasing sterilized males [3,4] and a growing awareness of the importance of understanding male reproductive success have led to repeated calls for addressing this neglected area in mosquito biology [5,6].

The mosquitoes that vector human malaria, the Anophelines, tend to mate in swarms that can vary from twenty to thousands of individuals and are usually extremely male-biased [7,8]. Swarms are initiated by males just before dusk and usually last less than 30 minutes, during which approaching females are rapidly mated [9,10]. Laboratory experiments with *Anopheles gambiae *[11] and *Anopheles culicifacies *[12] have confirmed that very little mating occurs after the first hour of darkness. Following insemination females leave the swarm, whereas males often return [10]. Females store the sperm in their single spermatheca and are capable of producing as many as nine clutches without remating [*An. stephensi*; [13]]. Although multiple mating is common in the laboratory [14,15], molecular studies suggest that females rarely mate more than once in the field [16,17]. How often males mate in the field is unknown, but laboratory studies have found substantial variation in male reproductive success, with many males failing to mate while others mate several times [18,19]. *Anopheles stephensi *males can inseminate up to four females over two nights [19] and *A. gambiae *males can inseminate up to 10 females per night [20].

Factors that influence male reproductive success in Anopheline mosquitoes include body size and age. In *A. gambiae *and *Anopheles freeborni*, large males are better at acquiring mates [10,21] although this is not always the case in *A. gambiae *[22]. In addition, Okanda *et al *[20] showed that *A. gambiae *males prefer larger, more fecund females. Male mate choice or assortative mating has foiled the release of sterile males in the past [4] and may compromise future release of transgenic mosquitoes [23]. With respect to male age, in *A. gambiae s.l*. and *Anopheles culifacies*, a virgin male's ability to inseminate a female peaks about seven days after emergence [19,24,25]. In contrast, two days old *A. gambiae *males were most likely to induce female oviposition [26]. These conflicting studies suggest that different measures of male fitness, e.g. the proportion of inseminated vs. ovipositing females, can give different results.

Other factors, and in particular genetic factors, influencing male reproductive success are virtually unknown. Yet, it is the genetic variation of reproductive success (and its genetic correlation with other life history traits or with resistance to malaria) that will determine the outcome of releasing transgenic or sterile males for malaria control. The objectives of the present study were (i) to test whether the variation in male reproductive success has a genetic component, and (ii) to test whether male reproductive success is genetically correlated with age of emergence and a measure of body size (i.e. wing length). For comparison and to check whether our population of *A. gambiae *contains quantitative genetic variation, full sib heritabilities of the age of emergence and wing length were also calculated.

In this study, male reproductive success was partitioned into two separate components, referred to as (i) mating success and (ii) fertilization success. The first component, mating success, is determined by a male's ability to catch a female and transfer enough sperm to her spermatheca to induce her to oviposit. Hence male mating success was measured as the proportion of available females that oviposit (binomial scale) or the mean number of eggs produced per available female (normal scale). The second component, fertilization success, is determined by the quality of a male's sperm and its ability to produce viable larvae and, because we can only score it in ovipositing females, depends on the first component (i.e. a male may transfer perfectly good sperm but we cannot know this if his partner fails to oviposit). Hence fertilization success was measured as the proportion of ovipositing females that produced viable larvae (binomial scale) or the mean number of larvae produced per ovipositing female (normal scale). Finally, the product of these two components provides an estimate of overall reproductive success measured either as the proportion of available females that produced larvae (binomial scale) or the mean number of larvae per available female (normal scale). While only overall reproductive success is relevant to evolutionary trajectories, studying the mechanisms underlying its constituent components and their potential for genetic variation and manipulation will undoubtedly become necessary following the release of transgenic Anopheline males. This study is the first attempt to measure quantitative genetic variation for male reproductive success in *An. gambiae*.

## Methods

The G3 colony of *A. gambiae sensu stricto *(courtesy of Christopher Christophides, Imperial College) was used in this experiment. Mosquitoes were kept in an insectary at 26°C, 70% relative humidity and 12 hours day and night cycle with 60 minutes of simulated dawn and dusk. Larvae were fed 0.03, 0.04, 0.08, 0.16, 0.32 and 0.60 mg of Tetramin per individual on days 1, 2, 3, 4, 5, 6 and onward, respectively, and were reared at a standard density to avoid density-induced variation in adult body size (see below). Adults were given *ad libitum *access to 8% sugar solution except for the 12 hours before a blood meal. Mosquitoes were mated in 20 cm cubic plexiglass cages with a mesh window that allowed blood feeding. All females were blood fed on the arms and legs of MJV in the evening after the lights were switched off, as this is the time when Anopheline females become active and seek out human hosts. In the field, *A. gambiae *females often use the first blood meal to supplement low teneral reserves and the second blood meal to produce eggs [27]. So that females had sufficient resources for producing eggs, they were allowed to feed twice (once before and once after mating, see below).

**Figure 1:**
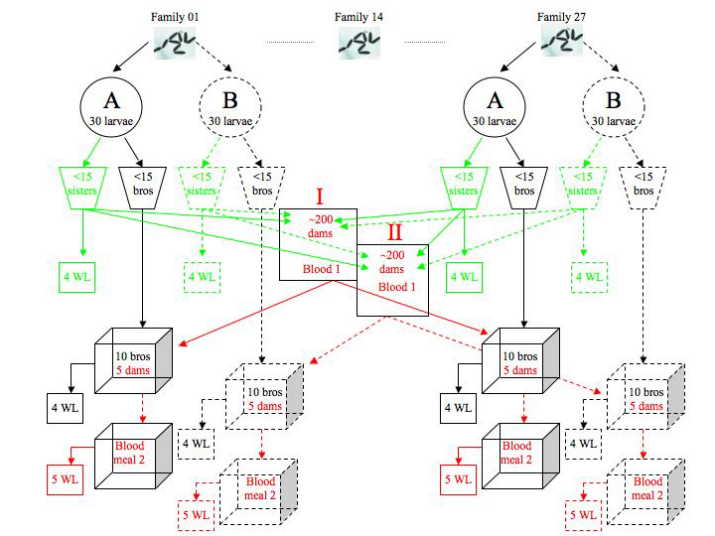
**Experimental Design**. Experimental design is shown for two of the 27 full sib families (see Methods for full explanation). Each family started with > 60 recently hatched larvae split into two blocks (A and B) of 30 larvae that produced roughly equal numbers (< 15) of brothers (bros) and sisters. For each of the 54 combinations of full sib family*block, four sisters were randomly selected for wing length (WL) measurements and the remaining females were split between two cages (I and II) where the females (now dams) received their first blood meal (Blood 1). For each of the 27 sire families in block A, ten brothers were randomly selected and combined with five randomly selected blood-fed dams from cage I. These mixed-sex swarms were allowed to mate for 24 hours, after which the males were removed and the dams were given their second blood meal. Two days later this process was repeated for the 27 sire families in block B and the blood-fed dams from cage II. For each of the 54 mating cages, the wing length of four randomly selected brothers and all 5 dams were measured. Blocks A and B are shown with solid and stippled lines, whereas brothers, sisters and dams are colour-coded black, green and red.

Previously, a group of 180 blood-fed females and 180 males had been given the opportunity to mate for 24 hours. After removing the males, the females were given a second blood meal before being transferred to individual oviposition cups. Of the 180 females, 64 laid eggs and 27 of these clutches produced at least 60 larvae. The larvae from these 27 families were the foundation of the present experiment (Figure 1). Although the percentage of multiply inseminated *An. gambiae *females can reach 24% in the laboratory [15] and therefore some of the 27 families may contain a mixture of full and half siblings, for convenience these families will hereafter be referred to as full sib families.

So that larval rearing density was standardized, 60 full sib larvae were haphazardly selected from each of the 27 families within one day of hatching. So that environmental and genetic effects could be distinguished, each family of 60 larvae was split into two blocks of 30 larvae (blocks A and B). Each block was reared in a Petri dish (ϕ = 86 mm) containing 10 ml of distilled water on the standard Tetramin diet (see above). Pupae were separated by sex (so that adults were virgins), placed into 500 ml plastic cups covered with mesh, and scanned daily to determine the age of emergence. For each of the 54 male-containing cups, the brothers were captured individually and the order of capture was used to randomly assign ten brothers to be used as sires in the mating assay. For example, cup 1 contained 11 brothers and the random sample function in R randomly selected brothers 2 to 11 from the capture sequence. The first male captured from cup 1 was set aside as a spare in case one of his brothers died prior to the mating assay. For each of the 54 female-containing cups, the same protocol randomly assigned four sisters to be frozen and measured for wing length, whilst the remaining sisters were evenly split among the two cages (I and II). Hence each of the two cages contained sisters from the same 27 families for a total of ~200 females per cage. These 400 females were used as dams in the mating assay and they were pooled for blood feeding, as it is much easier to feed blood-feed two cages than 54 cups. By pooling the dams their family identity was lost, but this did not matter as they were randomly assigned to the 54 groups of brothers. Due to shortage of cages it was not possible to mate all 54 groups on the same day, so blocks A and B were staggered in time. The 27 full sib sire families in block A and their randomly selected dams were processed first (as explained below) and, two days later, the whole sequence of events was repeated for block B.

The 200 females in cage I were blood fed 3 days after emergence for one hour. The next day 135 blood-fed females were captured and sequentially assigned to one of 27 plastic cups (500 ml), so that the 1^st^, 28^th^, 55^th^, 82^nd ^and 109^th ^captured female were all assigned to cup 1, the 2^nd^, 29^th^, 56^th^, 83^rd ^and 110^th ^captured female were assigned to cup 2, etc. These 27 cups, each containing five blood-fed females, were randomly assigned to the 27 sire families in block A. For each of these 27 sire families in block A, the 10 brothers were stored together in one of 27 mating cages. Five days after emergence, the sexes were brought together between 16:00 and 17:00. The males were removed 24 hours later and frozen for wing length measurements. The females were left in the cages and given their second blood meal that same night. The 27 cages were randomly assigned to one of four limbs (left or right forearm, left or right calf) and 7 feeding slots (21:30, 22:00, 22:30, 23:00, 23:30, 00:00, 00:30) and females were allowed to blood feed for 20 minutes.

Female mosquitoes rarely move once they start imbibing blood as long as the person feeding the mosquitoes stays very still (MJV personal observation). Once the females are close to fully fed they start pressing out serum, which makes a concentrated streak on the bottom of the cage. An estimate of the number of fully fed females following the second blood meal was obtained by placing a piece of paper on the bottom of each cage to catch this excreted serum. Mosquitoes were blood fed at night in the dark; this unorthodox method was used to avoid having to turn on the lights and disturb the natural order of things. The number of blood spots was never greater than the number of females present in the cage and there was rarely any overlap between adjacent bloodspots, suggesting that this method gave a reasonable estimate of the number of fully fed females.

The day after the second blood meal, females were transferred to individual 'oviposition cups': a 500 ml plastic cup covered with mesh, containing a small Petri dish (ϕ = 48 mm) filled with water and lined with Whatman filter paper for oviposition. For one week, these oviposition cups were checked every day to determine if the female had laid eggs. Eggs were monitored for hatching for a week. It was observed that females laid both black and white eggs and so these were counted separately. One week after the second blood meal, all females were frozen. Over the next three weeks, their abdomens were dissected for any remaining eggs, their wing lengths were measured and their spermatheca were dissected and checked for sperm. However, no sperm was found in the spermatheca for a substantial number of females that had produced larvae, so this phenotype was not a reliable measure of insemination. Hence it was not possible to separate the proportion of inseminated females from the proportion of ovipositing females, so these two components were combined into a single component of mating success.

Hence from the time of their emergence, females in block A were first blood-fed on day 2, mated with four-day old virgin males on day 4, blood-fed a second time on day 5 and then allowed to oviposit from days 6 to 13. For the males and females in block B this sequence was offset by two days so that block B females were first blood-fed on day 4, mated with six-day old virgin males on day 6, blood-fed a second time on day 7 and then allowed to oviposit from days 8 to 15.

For all sires, sisters and dams the length of both wings from the wing tip to the distal end of the alula was measured using a dissecting scope with an ocular micrometer (20× magnification). In this study, wing length was used as an estimate of body size as the correlation between wing length and body mass has been confirmed on numerous occasions [28,29].

### Statistical methods

For the 54 mating cages, the measures of male reproductive success were (1) the proportion of ovipositing females, (2) the proportion of ovipositing females that produced larvae, (3) the proportion of females that produced larvae, (4) the mean number of oviposited eggs per female (i.e. does not include retained eggs), (5) the mean number of larvae per ovipositing female and (6) the mean number of larvae per female. Those females that died during the first three days of oviposition were excluded so that the data were not biased by mortality. All means are given with their standard errors.

The generalized linear model function in R (glm) with binomial errors was used to model proportions (traits 1, 2 and 3) as a function of the factors sire family and block. The design precluded testing the sire family*block interaction term. The statistical significance of each factor was determined by comparing the main effects model (containing both factors) to a single factor model (containing either sire family or block) and comparing the change in the degrees of freedom (Δdf) and the change in deviance (Δdev) to a χ^2 ^distribution. For all three traits, the minimal adequate model (containing only factors that were statistically significant) fit the data well (i.e. residual degrees of freedom ≈ residual deviance).

The linear model function in R (lm) was used to model counts (traits 4, 5 and 6) as a function of the factors sire family and block. Again it was not possible to test the sire family*block interaction term, and the statistical significance of factors was determined via comparison of nested models (as for the proportion data above). The data fit the normal distribution reasonably well, because each of the 54 data points represents the mean number of oviposited eggs and larvae produced by the five females in the cage (or by the number of ovipositing females in the cage in the case of trait 5).

In addition, because individual wing length and fecundity estimates (laid + retained eggs) were obtained for most of the 270 dams included in the mating experiment, the lm function in R was used to test whether sire family affected female fecundity regardless of whether she laid the eggs or not while also controlling statistically for wing length and the number of females that fed to repletion following the second blood meal (i.e. the number of blood spots on the paper on the bottom of the cage).

The nonlinear and linear mixed effects models function in R (nlme) was used to estimate the variance component due to differences among sire families and the variance component due to differences between blocks of brothers (A and B) within sire families. These among (σ_A_^2^) and within (σ_W_^2^) sire family components were used to calculate the repeatability, t = σ_A_^2^/(σ_A_^2 ^+ σ_W_^2^) for all six traits [30]. Although the 'among sire family' variance contains a genetic component, these data cannot be used to estimate heritabilities of traits 1 to 6, because they represent the pooled efforts of ten brothers, whereas an estimate of the heritability of these traits would require estimates of individual male reproductive success.

For the age of emergence and wing length, the nlme function in R was used to estimate the variance components due to differences among full sib families, among blocks within families and among individuals within blocks and to calculate broad sense heritabilities [30]. Sex was included as a fixed factor, and family and block as random factors with the latter nested in the former. The variance component analysis for wing length also included a random factor for individual (nested within block), because there were two estimates of wing length for each individual. For calculating heritabilities for the age of emergence and wing length, families were assumed to contain only full siblings, and separate estimates were obtained for males and females. Statistical significance of fixed and random factors was determined by comparing nested models. The maximum likelihood option in the nlme function was used as this allows comparison of models with different fixed effects structures (i.e. between models with or without 'sex').

## Results

### Summary information

Of the 270 females, 95% survived (256/270) the first three days of oviposition, and 48% of these survivors (123/256) laid a total of 17,417 eggs of which 87% were black (15,101) and the remainder (2,316) were white. Of the 123 females that laid eggs, 75 laid black eggs, two laid white eggs and 46 individuals laid both black and white eggs. Of the 123 females that laid eggs, 68% of these ovipositing females (84/123) produced 8,495 larvae. For the 84 females that produced larvae, the number of larvae increased with the number of black eggs (multiple regression: slope = 0.84 ± 0.052 larvae/black egg, F_1,81 _= 295.18, p < 0.001) but was not significantly related to the number of white eggs (slope = -0.64 ± 0.418 larvae/white egg, F_1,81 _= 2.36, p = 0.128). The numbers of white and black eggs themselves were not correlated (r = 0.03, t = 0.49, df = 268, p = 0.621), so that they could be used as independent factors.

### Independence of female body size with respect to the 54 mating cages

As a check on the protocol of randomly assigning dams to the full sib sire families an ANOVA tested whether there was a significant difference in dam wing length among the 54 cages. There was no significant difference in dam wing length (F_53,190 _= 1.04, p = 0.408) among the 54 cages, indicating that the randomization protocol had worked. In addition, the difference in the mean wing length between ovipositing (3.06 ± 0.016 mm) and non-ovipositing (3.06 ± 0.015 mm) females was not significantly different (t = 0.15, df = 241, p = 0.879), indicating that males had no preference for larger or smaller females.

### Sire family effects on traits 1, 2 and 3

**Figure 2:**
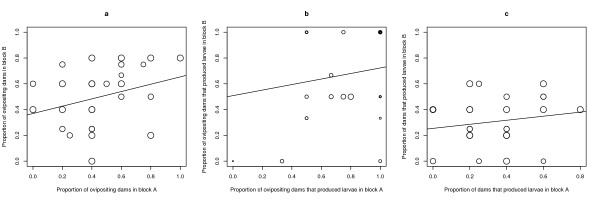
**Scatter plots of male fitness traits 1, 2 and 3**. Scatter plots of 27 sire families in blocks A and B for (a) the proportion of ovipositing dams (r = 0.32, df = 25, p = 0.102), (b) proportion of ovipositing dams that produced larvae (r = 0.42, df = 22, p = 0.040) and (c) proportion of dams that produced larvae (r = 0.17, df = 25, p = 0.383). The sizes of the data points are proportional to the geometric mean of the number of dams in the denominator of the trait of interest in blocks A and B. Shown are the lines of best fit.

For the 27 sire families, the proportion of ovipositing females ranged from 0.20 to 0.90 (Figure 2a). There was a significant effect of sire family (Δdf = 26, Δdev = 39.60, p = 0.043), but no effect of block (Δdf = 1, Δdev = 0.75, p = 0.388). The proportion of ovipositing females that produced larvae varied between 0.00 and 1.00 (Figure 2b). There was a significant effect of sire family (Δdf = 23, Δdev = 37.68, p = 0.028), but no effect of block (Δdf = 1, Δdev = 2.12, p = 0.145). The proportion of females that produced larvae ranged from 0.00 to 0.60 (Figure 2c). There was no effect of sire family (Δdf = 26, Δdev = 27.512, p = 0.383) or block (Δdf = 1, Δdev = 0.2090, p = 0.648). The repeatabilities of these three traits were 0.32, 0.39, and 0.19, respectively. These three components of male reproductive success were not significantly correlated with either male age of emergence or male wing length (Table 1).

**Table 1:** Correlation matrix for male age, wing length and six fitness traits

	age	wing	trait 1	trait 2	trait 3	trait 4	trait 5	trait 6
age	***	0.154	0.038	-0.091	0.097	0.022	-0.272	-0.130
wing	0.444	***	-0.103	0.016	-0.136	-0.027	0.068	-0.056
trait 1	0.850	0.610	***	-0.292	0.633	0.907	-0.227	0.551
trait 2	0.653	0.935	0.139	***	0.479	-0.331	0.677	0.298
trait 3	0.630	0.498	0.000	0.012	***	0.504	0.263	0.748
trait 4	0.914	0.894	0.000	0.091	0.007	***	-0.066	0.622
trait 5	0.170	0.737	0.255	0.000	0.184	0.745	***	0.602
trait 6	0.517	0.783	0.003	0.131	0.000	0.001	0.001	***

Correlation matrix for male age at emergence (age), male wing length (wing), proportion of ovipositing females (trait 1), proportion of ovipositing females that produced larvae (trait 2), proportion of females that produced larvae (trait 3), eggs per female (trait 4), larvae produced per ovipositing female (trait 5) and larvae produced per female (trait 6). Pearson's r correlations and the associated p-values are given above and below the diagonal, respectively. The bold type indicates statistical significance.

### Sire family effects on traits 4, 5 and 6

**Figure 3:**
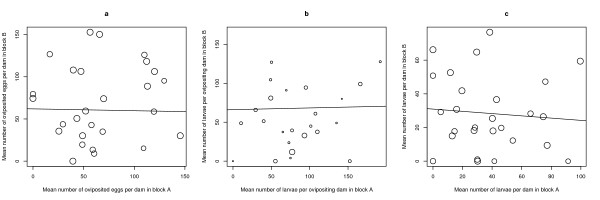
**Scatter plots of male fitness traits 4, 5 and 6**. Scatter plots of 27 sire families in blocks A and B for (a) mean number of oviposited eggs per dam (r = 0.09, df = 25, p = 0.67), (b) mean number of larvae per ovipositing dam (r = 0.25, df = 22, p = 0.241) and (c) the mean number of larvae per dam (r = -0.084, df = 25, p = 0.676). The sizes of the data points are proportional to the geometric mean of the number of dams in the denominator of the trait of interest in blocks A and B. Shown are the lines of best fit.

For the 27 sire families, the mean number of oviposited eggs per female ranged from 19.7 to 117.1 (Figure 3a). There was no effect of sire family (F_26,26 _= 1.19, p = 0.331) or block (F_1,26 _= 0.04, p = 0.837). The mean number of larvae per ovipositing female ranged from 0.0 to 165.5 (Figure 3b) with no effect of sire family (F_26,26 _= 1.78, p = 0.083). Block was significant (F_1,26 _= 7.06, p = 0.014), with block A (83.6 ± 9.08) producing more larvae per ovipositing female, on average, than block B (60.1 ± 8.91). Finally, the number of larvae per female ranged from 0.0 to 79.5 (Figure 3c) with no effect of sire family (F_26,26 _= 0.85, p = 0.661) or block (F_1,26 _= 1.63, p = 0.214). The repeatabilities of these three traits were 0.104, 0.140 and 0.000, respectively. Again there was no correlation between these three components of male reproductive success and male age or male wing length (Table 1).

### Female body size and blood-feeding effects on female fecundity

While the above analyses were based on the average values of the five females per cage (or the number of ovipositing females per cage), the next analyses are based on individual females. In addition to the 17,417 eggs that were laid, the 256 surviving females produced another 22,361 eggs that were retained in the abdomen. The mean fecundity (i.e. laid + retained eggs), including three females that produced no eggs, was 155.4 ± 4.16 eggs per surviving female (n = 256). The blood spots on the paper indicated that 74% of the females (190/256) fed to repletion during the second blood meal. Female wing length (F_1,218 _= 64.27, p < 0.001; no wing lengths for nine individuals, so n = 247) and the number of fully fed females per cage (F_1,218 _= 11.85, p < 0.001) increased female fecundity, but sire family had no effect (F_26,218 _= 1.12, p = 0.325). The minimal adequate model with wing length (b_1 _= 177.9 ± 21.98 eggs/mm) and the number of fully fed females following the second blood meal (b_2 _= 14.6 ± 3.58 eggs/second blood meal) accounted for 19% and 4% of the variation in female fecundity, respectively.

### Heritability of the age of emergence and wing length

The mean age of emergence for females (10.2 ± 0.09 days) was similar to that of males (10.1 ± 0.09 days; χ^2 ^= 1.95, p = 0.162). Differences among families (14%; χ^2 ^= 34.92, p < 0.001) and individuals (86%) accounted for a significant portion of the total variance in the age of emergence but differences between the two blocks did not (0%; χ^2 ^= 0.00, p = 1.00). The full sib heritability of the age of emergence was 0.32 in males and 0.24 in females. The mean wing length for females (3.04 ± 0.020 mm) was significantly larger than that of males (2.92 ± 0.020 mm; χ^2 ^= 73.82, p <0.001). Differences among families (29%; χ^2 ^= 93.06, p < 0.001) and individuals (69%; χ^2 ^= 1500.80, p < 0.001) accounted for a significant portion of the total variance in wing length but differences between the two blocks (1%; χ^2 ^= 0.14, p = 0.709) and between the left and right wing (1%) did not. The full sib heritability of wing length was 0.62 in males and 0.46 in females.

## Discussion

For the binomially distributed measures of male reproductive success, there was a significant effect of sire family on mating success (proportion of ovipositing females) and fertilization success (proportion of ovipositing females that produced larvae), but not on overall reproductive success (proportion of females that produced larvae). Thus, while the two components of male reproductive success (as defined in this study) – mating success and fertilization success – had a genetic basis, the overall reproductive success (which is relevant for evolutionary trajectories) did not. In contrast, we found no effect of sire family on any of the normally distributed measures of male reproductive success (number of oviposited eggs per female, the number of larvae per ovipositing female or the number of larvae per female). This discrepancy between the binomially and normally distributed measures of male reproductive success is easily reconciled by considering what mechanisms operating in which sex control the various stages of reproduction. For example, while products in the male ejaculate may induce a female to oviposit (see below), the number of eggs depends on the blood meal size and female body size, both of which are more likely to be under female control [31]

The repeatabilities of the proportion of ovipositing females (0.32), the proportion of ovipositing females that produced larvae (0.39), and the proportion of females that produced larvae (0.19) provide quantitative estimates of the covariance in these traits among groups of brothers. Although it is not possible to calculate the heritability of male reproductive success from this study (as this requires measures on individual males) it is useful to speculate on the factors that affect the repeatability of these traits, as these factors will also affect their heritabilities. The repeatability of a trait depends on its variance; the variance of a proportion, V(ρ) = ρ *(1 - ρ)/n (where ρ is the proportion of successes in n trials) reaches a maximum when ρ = 0.5. For example, male reproductive success in this laboratory colony increases with swarm size; less than 10% of females produce larvae when the swarm size is 5 males: 5 females, whereas 40% of females produce larvae when the swarm size is 30 males: 30 females (Voordouw & Koella; unpublished data), hence the repeatability or heritability of this trait will be higher for the larger swarm size. The repeatability also depends on the frequency of multiple mating by the mothers of the sire families, because the genetic covariance among full sib brothers is twice that of half sib brothers. Finally, the repeatability also depends on the degree to which oviposition is under female, rather than male, control and the variance in female oviposition behavior. The present study shows that there is some male control over female oviposition, but only if females are given a suitable environment in which they are willing to lay eggs.

What mechanisms could account for the variation in male reproductive success in our population of *A. gambiae*? Early work on *A. gambiae *[32,33] and *Aedes aegypti *[34] showed that fluids from the male accessory glands (MAGS) control female monogamy and oviposition. Later work found that a sperm-filled spermatheca [15] rather than MAGS [35] induces female oviposition behavior. Genetic differences in the size of the male's ejaculate may therefore influence a female's probability to lay eggs. The recent discoveries of polymorphic sperm in *A. gambiae *and that long sperm are more likely to make it into the spermatheca [36] suggest that heritable differences in sperm length, as found in other insects [37], might play a role in male fertilization success. In *Drosophila melanogaster*, accessory gland proteins in the male ejaculate induce ovulation before sperm storage has been completed resulting in inefficient fertilization and a lower hatching success than mutants that lack these proteins [38]. Hence, a number of potential mechanisms may account for the genetic variation in male mating and fertilization success observed in this study.

The probability of detecting a significant sire effect on our measures of reproductive success is compromised by the fact that so much of the genetic and environmental variance in these traits is under female control. For this reason, female effects were controlled for as much as possible by (1) randomly assigning females to the mating cages and (2) standardizing larval diet and larval density to minimize variation in female body size, blood meal size and fecundity. The fact that there was no significant difference in female wing length among the 54 cages indicated that the randomization protocol was successful and that the covariance in the normally distributed measures of male reproductive success (traits 4, 5 and 6) among full sib blocks was not confounded by mean differences in female body size. Of course it is impossible to completely eliminate all variation, and differences in wing length among females within a cage still accounted for ~20% of the variation in total fecundity (laid + retained eggs). More importantly, after controlling statistically for female body size and the number of females feeding to repletion on the second blood meal, there was no effect of sire family on female fecundity.

Another important female factor is her access to blood resources for producing eggs. To minimize this potential source of error, females were given ample time to blood feed (60 and 20 minutes for the first and second blood meal, respectively), only blood-fed females were included in the mating cages and these females were given a second blood-feeding opportunity after mating. The blood spots on the paper suggest that 74% of females fed to repletion during the second blood meal. Because females only press out the serum when they are close to being full this suggests that at least 74% of females took a full second blood meal. Further evidence that females obtained enough blood for egg production was that the average fecundity was high (155 eggs/female) and that the vast majority (253/256) contained at least some eggs.

One major shortcoming of this experiment is that, because the spermatheca from frozen females did not provide reliable estimates of whether the female had been inseminated or not, we were unable to separate insemination from oviposition success. Hence of the 130 females that contained eggs, but did not lay them, it is not known whether they were not inseminated or they were inseminated but judged the oviposition cups to be an inadequate environment for laying eggs. It was recently shown for the Keele strain of *An. gambiae *that 25% of females that appear to have all the requirements for oviposition (two blood meals and sperm in their spermatheca) failed to lay any eggs over a period of 10 days (Voordouw, Koella and Hurd; in review). In that experiment it was not clear whether the failure to oviposit was under male control (i.e. his ejaculate failed to signal to the female that she was mated) or female control (i.e. she received the male's signal but other cues such as a suitable oviposition environment were missing). Finally, almost a third of the females that laid eggs did not produce any larvae and again, it is not clear whether this was caused by poor quality sperm or by some sort of genetic or cytoplasmic incompatibility between males and females. A cross-factorial experimental design that mates 'a' sire families with each of 'b' dam families would allow one to estimate the paternal and maternal components of oviposition and hatch success and determine whether there are sire*dam interactions.

Previous studies have shown that egg laying is rare in unmated *A. gambiae *females [4.2%; [26]] and that most ovipositing females (> 95%) lay their eggs within 7 days of having access to an oviposition site [26]. In our study females laid white eggs that never produced larvae suggesting that these eggs were either unfertilized or were fertilized but failed to sclerotize and therefore did not hatch. Hence it is not clear whether these white eggs reflect aberrant female oviposition behavior or male fertility. However, this distinction does not affect the analysis of the proportion of ovipositing females (trait 1) as only two females exclusively laid white eggs (i.e. 90.7% of the white eggs were laid by females that also produced black eggs). In addition, we are confident that we observed almost all hatching events because 99% of *A. gambiae *larvae hatch within one week of oviposition [39]. The proportion of ovipositing females (0.48) fell within the observed range from two other *A. gambiae *mating studies (0.83 in [11], 0.26 in [25]) where the experimental conditions (2 males: 1 female sex ratio, 24 hours mating period) were comparable to our study.

Of the 15,101 black eggs that were laid, only 56% produced larvae. The hatching success in our study was similar to other lab colonies of *A. gambiae *[43 – 63%; [40]], but lower than that of field-collected individuals [86%; [39]], suggesting some inbreeding effects. In mosquitoes of the genus *Culex *and *Aedes*, low hatching success is commonly caused by *Wolbachia*, a maternally inherited bacteria that induces cytoplasmic incompatibility where sperm from infected males are unable to fertilize the eggs of uninfected females, however, this phenomenon is not known to occur in Anopheline mosquitoes [41].

There was no evidence that male age of emergence and body size were genetically correlated with any component of male reproductive success (Table 1). Our broad sense heritabilities for male age of emergence (0.32) and wing length (0.62) were highly significant (p < 0.001) and are conservative estimates if some of the families contained half siblings. Furthermore, our protocol or rearing 30 larvae in different Petri dishes for each of the 27 families accounts for less than 1% of the phenotypic variance in the age of emergence and wing length. Hence, it is unlikely that environmental or measurement error in these two traits obscured any potential genetic correlation with male reproductive success. Hence there was no evidence that males preferentially mated with larger and/or more fecund females as suggested by Okanda et al. [20].

The genetic architecture of female fitness and malaria resistance has understandably been the focus of much theoretical [42,43] and empirical work [40,44] given its epidemiological significance. However, as molecular biologists reveal the abundance of genes involved in protecting female mosquitoes from malaria parasites [45-48] and as others, using a variety of insects, demonstrate the ubiquity of trade-offs between immunity and other life history traits [49-51], there is a growing awareness that unless the expression of these anti-malarial genes is perfectly sex-linked, males will play a role in their evolution. Furthermore, the release of competitive, transgenic males in the field represents an opportunity for sexual selection on the male phenotype with potentially undesirable consequences. For example, in an artificial selection experiment with *Drosophila melanogaster*, lines with a 5 male: 1 female operational sex ratio evolved larger body size and increased adult survivorship relative to lines with a 1:1 sex ratio [52]. Similar developments in wild populations of *A. gambiae *would be disastrous, as long-lived, malaria-infected females would increase the probability of malaria transmission from mosquito to man. Hence despite the success of creating transgenic, malaria-resistant female mosquitoes [53,54], future research requires a shift towards males.

## Authors' contributions

MJV conceived the idea, ran the experiment and analyzed the data. MJV and JCK both designed the experiment, interpreted the data and wrote the paper.
